# Forests of the Night: Refugia of Genetic Diversity in Wild Tigers

**DOI:** 10.1371/journal.pgen.1000603

**Published:** 2009-08-14

**Authors:** Oliver A. Ryder

**Affiliations:** San Diego Zoo's Institute for Conservation Research, Escondido, California, United States of America; Stanford University, United States of America

Having amassed non-invasively collected samples from 73 tigers from 28 Indian reserves, Mondol, Karanth, and Ramakrishnan have published a comprehensive study of genetic variation in tigers in *PLoS Genetics* [1]. Incorporating information on mitochondrial haplotypes and microsatellite allelic diversity from previous studies, their additional data from the wild tiger populations in India demonstrate that the greatest extent of tiger genetic diversity resides in the Indian subcontinent. The expansion and refinement of knowledge pertaining to tiger phylogeography and population genetics comes at a time when small population vulnerabilities, poaching, and habitat loss conspire to produce an apocalypse for these charismatic cats.

**Image 1:**
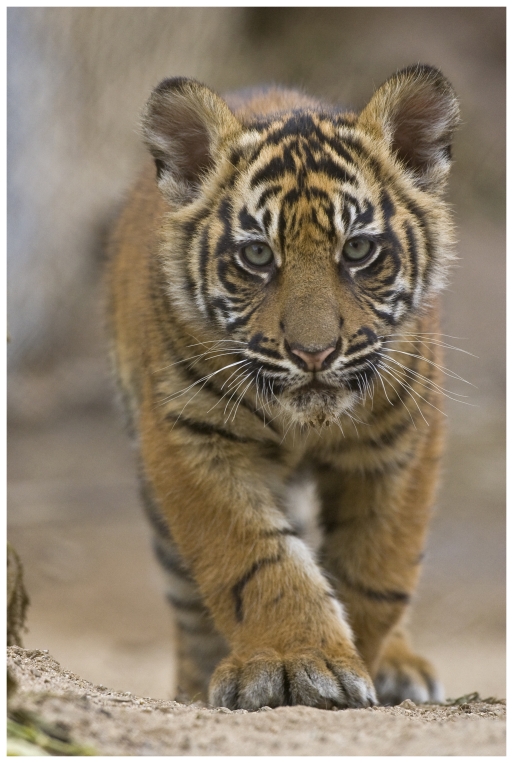
Sumatran Tiger cub. (Image: San Diego Zoo, http://www.sandiegozoo.org).

Those people providing for the future of tigers in the wild must rely on the protection of sufficient habitat and the maintenance of self-sustaining populations and the processes required to support them. Of the eight named subspecies of tigers that were present in the last century, only five survive, and one of these only in captivity. Many more tigers are held in captivity than remain in the wild. Cooperative management programs to preserve the genetic diversity of zoo-based tigers exist for the Sumatran, Amur, and Indochinese tigers within zoo associations in North America, Europe, and Asia. In a remarkable statistic, more tigers are owned by individuals or institutions that may not document pedigrees nor adopt other guidelines considered best practices than are managed in these conservation programs. The allure of tigers for magical or medicinal properties, for prestige, and as icons of beauty and wildness (Image 1) has contributed to their vulnerability.

As robust methods for assessing variation in the nuclear and mitochondrial genomes of tigers have been developed and as samples have been accumulated for study, the phylogeny of tigers, the knowledge of evolutionary divergence of extinct and surviving populations, and the depletion of genetic diversity of tigers across Asia have advanced [2]. The addition of new data on mitochondrial sequence data from wild Indian tigers considerably expands the mitochondrial haplotype network and reveals a larger genetic size of Indian tiger populations than previously documented. While the mitochondrial haplotype network of tiger populations that have been widely separated geographically in historic times reflects a relatively short evolutionary history, these data, combined with nuclear microsatellite analyses, support the recognition of evolutionary units for tiger conservation, including two lineages of Indochinese tigers previously considered as a single subspecies [2],[3].

Utilizing multiple approaches, analyses of microsatellite variation from the Indian tigers by Mondol et al. [1], as well as from Indochinese tigers studied by Luo et al. [2], suggests a decline in population numbers of Indian and Indochinese tigers of approximately 90% over approximately the same recent period (150–200 years). The greater extent of genetic variation remaining in Indian as contrasted with Indochinese tigers is a consequence of a smaller historical median effective population size of Indochinese tigers than tigers from central and south India.

Although phylogeographic studies indicate tigers expanded their range in the Pleistocene era from northern Indochina and southern China, based on their data and previous studies [2] Mondol et al. suggest that Indian tigers retain more than 60% of the species' genetic variability. India, though not the origin of tiger evolutionary diversification, can now be said to harbor the greatest extent of remaining genetic diversity. While the studies from S. J. O'Brien's lab have built an appreciation of the discrete divisions of extant tiger populations in the form of recognized subspecies, Mondol et al. suggest that, in consideration of their findings that 76% of the mitochondrial diversity and 63% of the total species' nuclear microsatellite diversity is present in Indian tiger populations, “subspecies-based conservation criteria are inappropriate.” The reservoir of tiger genetic diversity in India includes populations occurring in a wide diversity of habitat types, from the Himalayan foothills to the southern Indian tropical moist forests, Mondol et al. point out; they suggest that the notable ecological diversity of Indian tigers also recommends Indian tiger populations as a priority for conservation efforts. Their proposal merits serious consideration, but the realities of providing sufficient habitat for expanding tiger populations should also be calculated into global efforts for tiger conservation [4].

Numerous injuries and fatalities of humans by wild tigers and tigers who attack their caretakers attest to the recalcitrance of the tiger gene pool to domestication. Human agency has, however, resulted in significant admixture of evolutionary lineages of tigers; hybridization in captive tiger populations is widespread, with the exception of many of the zoo-based management programs [5]. Tiger-breeding facilities that produce tiger-derived products for the marketplace engender greater concern [6] and criticism [7] than exist for other endangered species, such as crocodilians. The development of databases of genotypes of wild tigers now facilitates the identification of admixed tigers and assists in the retention of genetic diversity of ecological and evolutionary subspecies that can reinforce the opportunities for linking *ex situ* and *in situ* tiger conservation efforts.

In the context of saving tigers in the differing physical environments—from moist tropical forests to subarctic taiga—and of divergent human cultural values of wildness and conservation, and in the face of the limited extent of suitable and safe wild habitat remaining to support tiger populations, it will be difficult to find a consensus for human interdiction that equally serves all tiger populations. Among the annals of conservation successes, in the face of human population growth and development over the last 60 years, India has produced a great accomplishment with its efforts to save tigers. Mondol et al. have elucidated that the evolutionary potential—in the form of genetic diversity—is available to sustain tigers into the future, if humankind chooses to do so.
